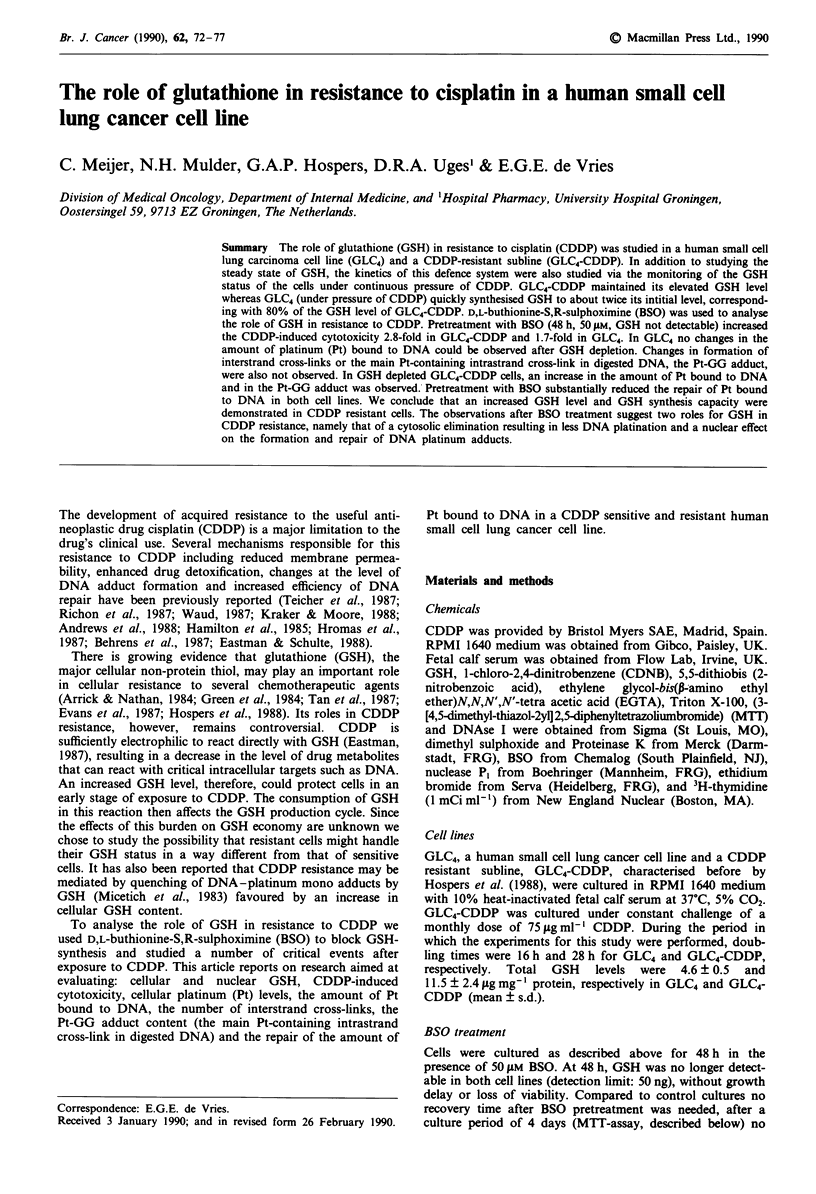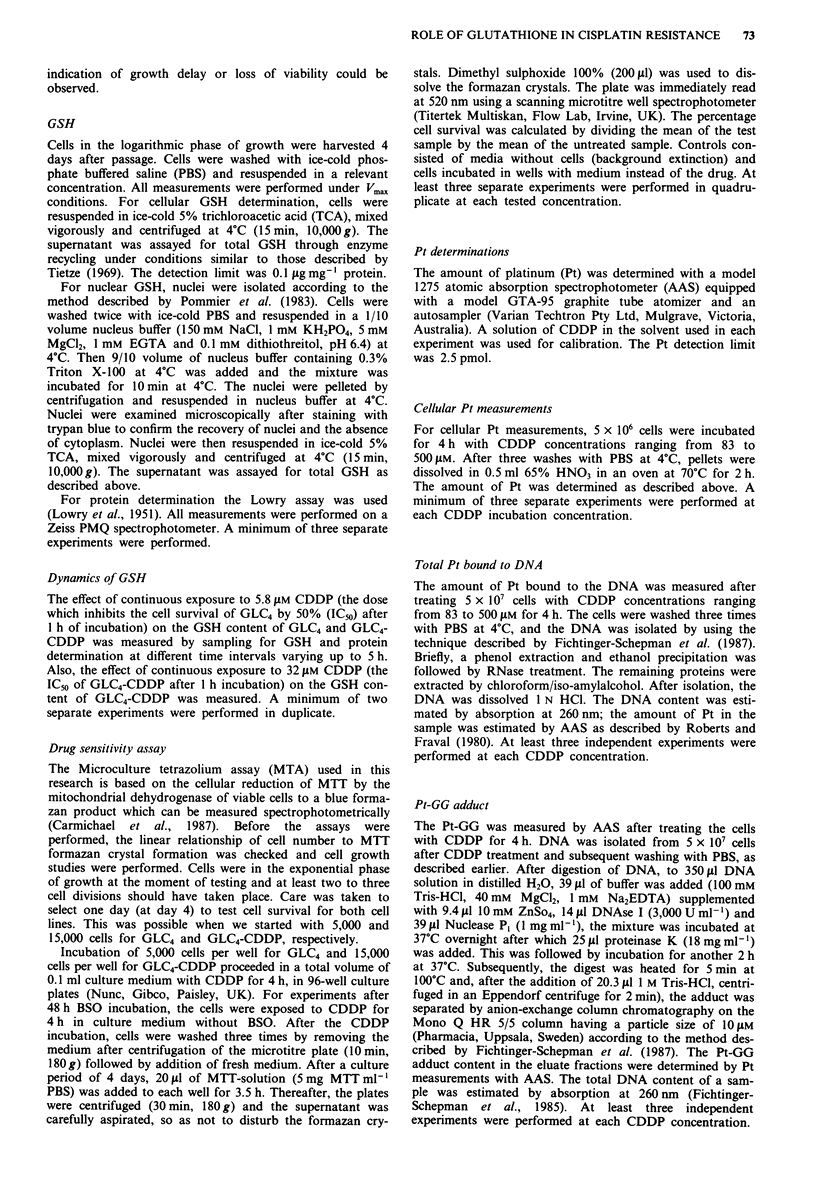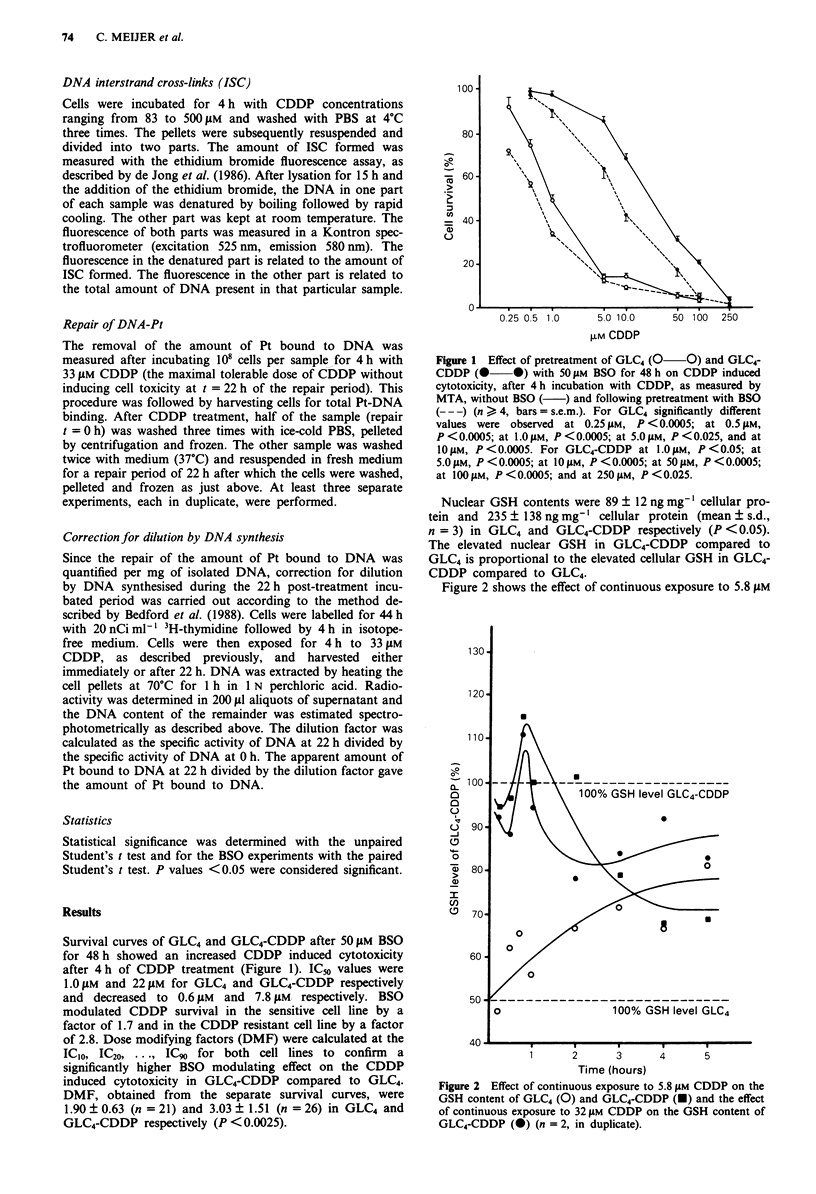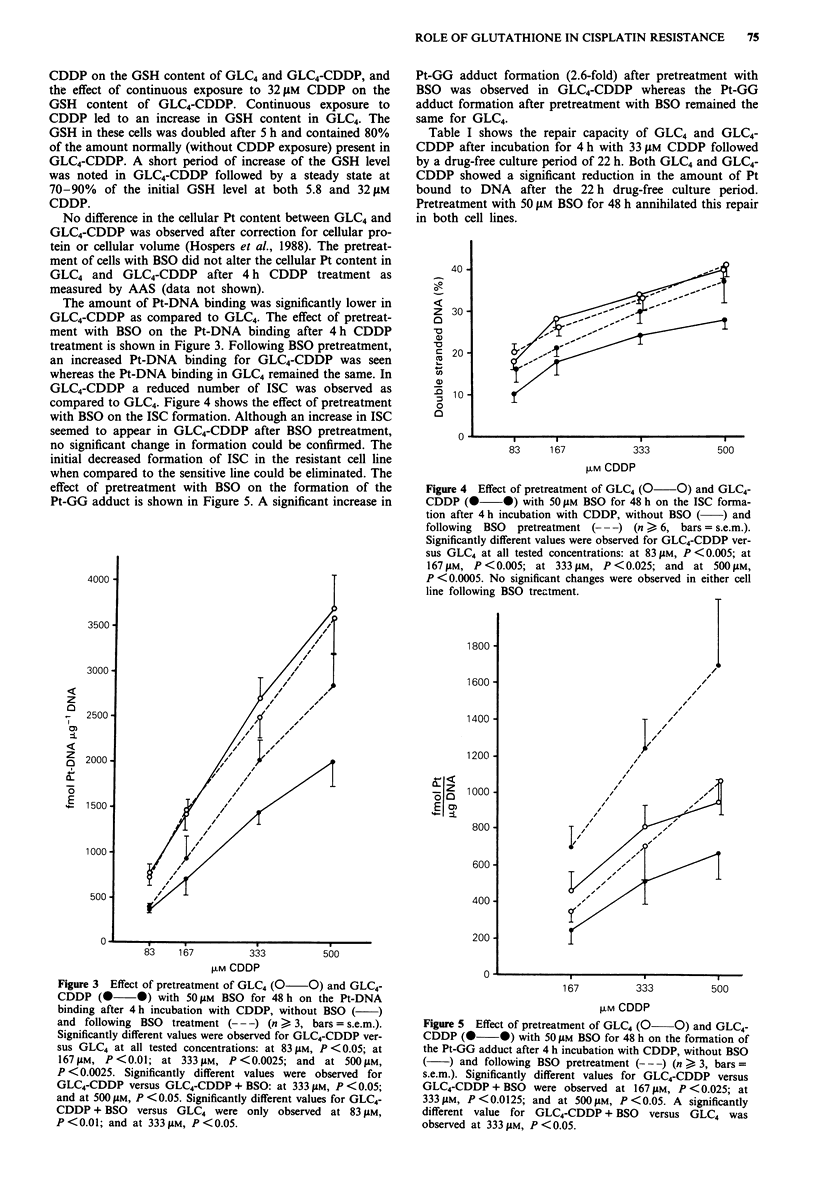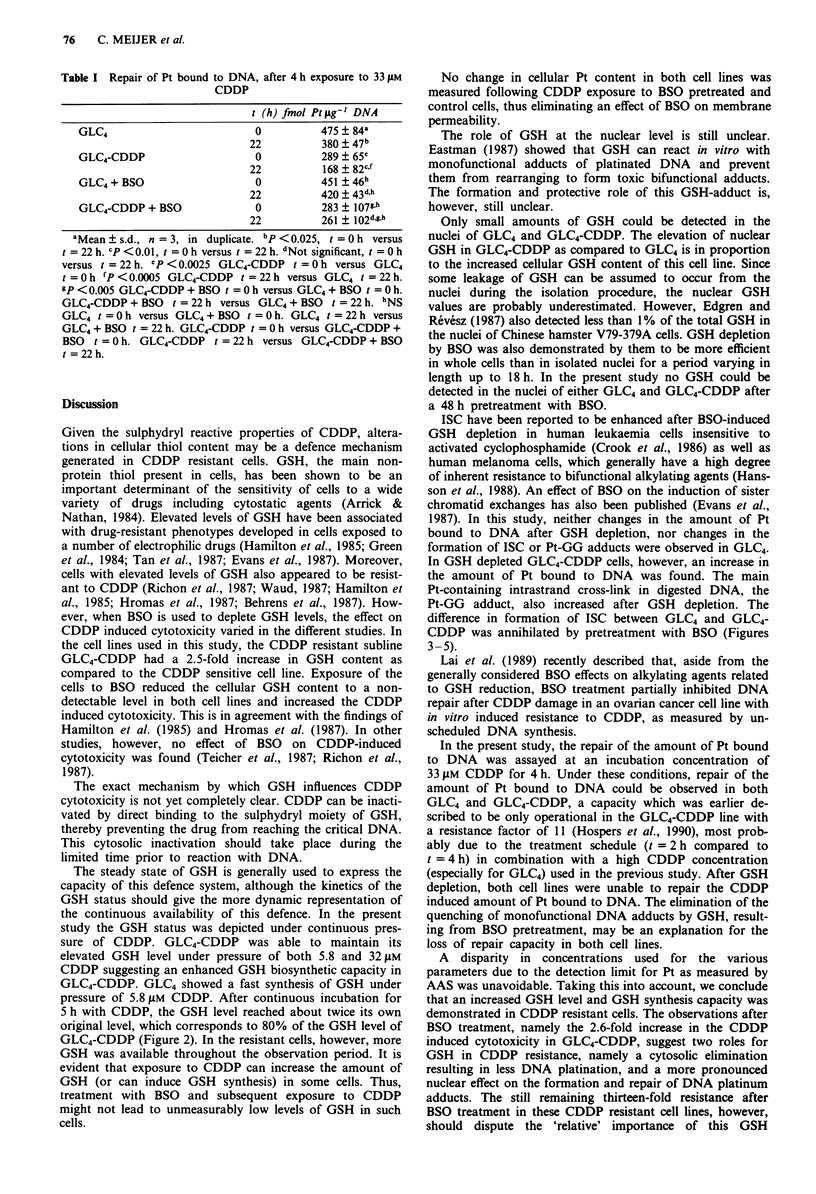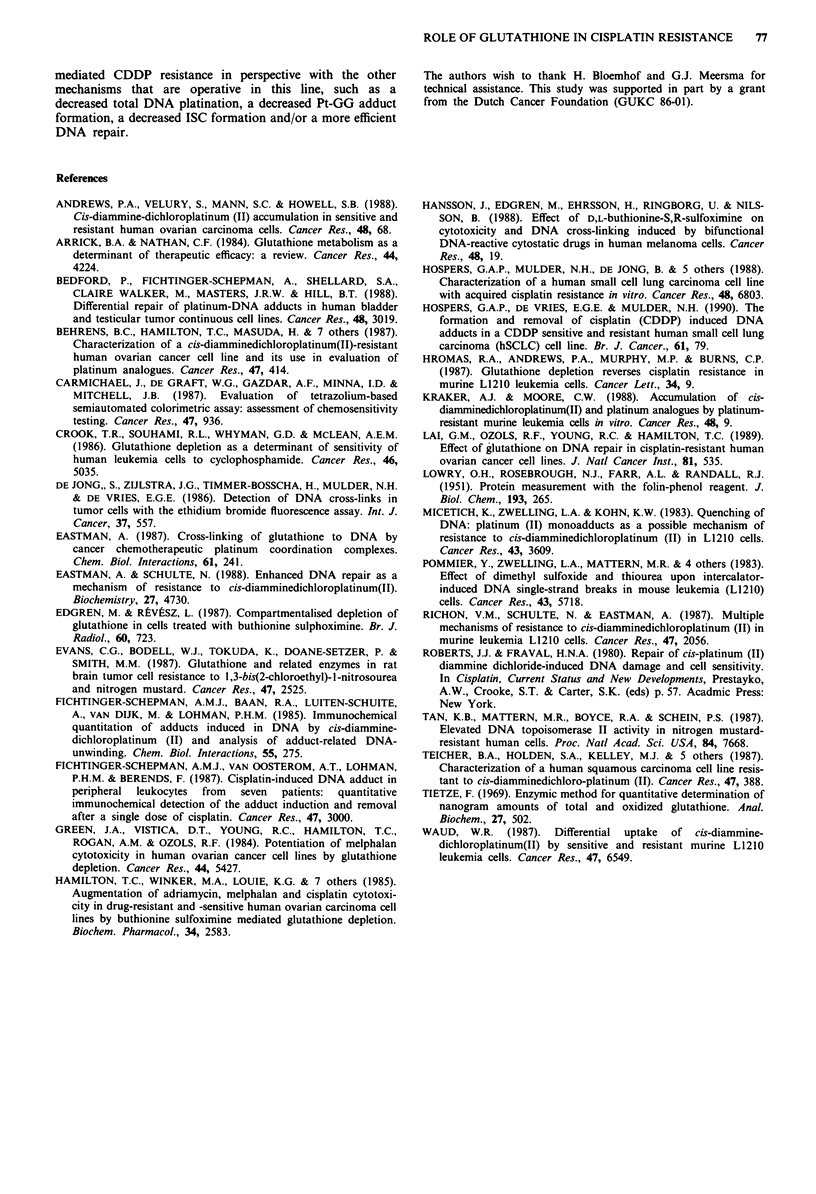# The role of glutathione in resistance to cisplatin in a human small cell lung cancer cell line.

**DOI:** 10.1038/bjc.1990.232

**Published:** 1990-07

**Authors:** C. Meijer, N. H. Mulder, G. A. Hospers, D. R. Uges, E. G. de Vries

**Affiliations:** Department of Internal Medicine, University Hospital Groningen, The Netherlands.

## Abstract

The role of glutathione (GSH) in resistance to cisplatin (CDDP) was studied in a human small cell lung carcinoma cell line (GLC4) and a CDDP-resistant subline (GLC4-CDDP). In addition to studying the steady state of GSH, the kinetics of this defence system were also studied via the monitoring of the GSH status of the cells under continuous pressure of CDDP. GLC4-CDDP maintained its elevated GSH level whereas GLC4 (under pressure of CDDP) quickly synthesised GSH to about twice its initial level, corresponding with 80% of the GSH level of GLC4-CDDP. D,L-buthionine-S,R-sulphoximine (BSO) was used to analyse the role of GSH in resistance to CDDP. Pretreatment with BSO (48 h, 50 microM, GSH not detectable) increased the CDDP-induced cytotoxicity 2.8-fold in GLC4-CDDP and 1.7-fold in GLC4. In GLC4 no changes in the amount of platinum (Pt) bound to DNA could be observed after GSH depletion. Changes in formation of interstrand cross-links or the main Pt-containing intrastrand cross-link in digested DNA, the Pt-GG adduct, were also not observed. In GSH depleted GLC4-CDDP cells, an increase in the amount of Pt bound to DNA and in the Pt-GG adduct was observed. Pretreatment with BSO substantially reduced the repair of Pt bound to DNA in both cell lines. We conclude that an increased GSH level and GSH synthesis capacity were demonstrated in CDDP resistant cells. The observations after BSO treatment suggest two roles for GSH in CDDP resistance, namely that of a cytosolic elimination resulting in less DNA platination and a nuclear effect on the formation and repair of DNA platinum adducts.


Br. J. Cancer (1990), 62, 72-77 © Macmillan Press Ltd., 1990

### The role of glutathione in resistance to cisplatin in a human small cell lung cancer cell line

C. Meijer, N.H. Mulder, G.A.P. Hospers, D.R.A. Uges[1] & E.G.E. de Vries

*Division of Medical Oncology, Department of Internal Medicine, and [1]Hospital Pharmacy, University Hospital Groningen, Oostersingel 59, 9713 EZ Groningen, The Netherlands.*

**Summary** The role of glutathione (GSH) in resistance to cisplatin (CDDP) was studied in a human small cell lung carcinoma cell line ($GLC_4$) and a CDDP-resistant subline ($GLC_4$-CDDP). In addition to studying the steady state of GSH, the kinetics of this defence system were also studied via the monitoring of the GSH status of the cells under continuous pressure of CDDP. $GLC_4$-CDDP maintained its elevated GSH level whereas $GLC_4$ (under pressure of CDDP) quickly synthesised GSH to about twice its intitial level, corresponding with 80% of the GSH level of $GLC_4$-CDDP. D,L-buthionine-S,R-sulphoximine (BSO) was used to analyse the role of GSH in resistance to CDDP. Pretreatment with BSO (48 h, 50 μM, GSH not detectable) increased the CDDP-induced cytotoxicity 2.8-fold in $GLC_4$-CDDP and 1.7-fold in $GLC_4$. In $GLC_4$ no changes in the amount of platinum (Pt) bound to DNA could be observed after GSH depletion. Changes in formation of interstrand cross-links or the main Pt-containing intrastrand cross-link in digested DNA, the Pt-GG adduct, were also not observed. In GSH depleted $GLC_4$-CDDP cells, an increase in the amount of Pt bound to DNA and in the Pt-GG adduct was observed. Pretreatment with BSO substantially reduced the repair of Pt bound to DNA in both cell lines. We conclude that an increased GSH level and GSH synthesis capacity were demonstrated in CDDP resistant cells. The observations after BSO treatment suggest two roles for GSH in CDDP resistance, namely that of a cytosolic elimination resulting in less DNA platination and a nuclear effect on the formation and repair of DNA platinum adducts.

The development of acquired resistance to the useful anti-neoplastic drug cisplatin (CDDP) is a major limitation to the drug's clinical use. Several mechanisms responsible for this resistance to CDDP including reduced membrane permeability, enhanced drug detoxification, changes at the level of DNA adduct formation and increased efficiency of DNA repair have been previously reported (Teicher *et al.*, 1987; Richon *et al.*, 1987; Waud, 1987; Kraker & Moore, 1988; Andrews *et al.*, 1988; Hamilton *et al.*, 1985; Hromas *et al.*, 1987; Behrens *et al.*, 1987; Eastman & Schulte, 1988).

There is growing evidence that glutathione (GSH), the major cellular non-protein thiol, may play an important role in cellular resistance to several chemotherapeutic agents (Arrick & Nathan, 1984; Green *et al.*, 1984; Tan *et al.*, 1987; Evans *et al.*, 1987; Hospers *et al.*, 1988). Its roles in CDDP resistance, however, remains controversial. CDDP is sufficiently electrophilic to react directly with GSH (Eastman, 1987), resulting in a decrease in the level of drug metabolites that can react with critical intracellular targets such as DNA. An increased GSH level, therefore, could protect cells in an early stage of exposure to CDDP. The consumption of GSH in this reaction then affects the GSH production cycle. Since the effects of this burden on GSH economy are unknown we chose to study the possibility that resistant cells might handle their GSH status in a way different from that of sensitive cells. It has also been reported that CDDP resistance may be mediated by quenching of DNA-platinum mono adducts by GSH (Micetich *et al.*, 1983) favoured by an increase in cellular GSH content.

To analyse the role of GSH in resistance to CDDP we used D,L-buthionine-S,R-sulphoximine (BSO) to block GSH-synthesis and studied a number of critical events after exposure to CDDP. This article reports on research aimed at evaluating: cellular and nuclear GSH, CDDP-induced cytotoxicity, cellular platinum (Pt) levels, the amount of Pt bound to DNA, the number of interstrand cross-links, the Pt-GG adduct content (the main Pt-containing intrastrand cross-link in digested DNA) and the repair of the amount of Pt bound to DNA in a CDDP sensitive and resistant human small cell lung cancer cell line.

Correspondence: E.G.E. de Vries.
Received 3 January 1990; and in revised form 26 February 1990.

### Materials and methods

### *Chemicals*

CDDP was provided by Bristol Myers SAE, Madrid, Spain. RPMI 1640 medium was obtained from Gibco, Paisley, UK. Fetal calf serum was obtained from Flow Lab, Irvine, UK. GSH, 1-chloro-2,4-dinitrobenzene (CDNB), 5,5-dithiobis (2-nitrobenzoic acid), ethylene glycol-*bis*(β-amino ethyl ether)*N*,*N*,*N*′,*N*′-tetra acetic acid (EGTA), Triton X-100, (3-[4,5-dimethyl-thiazol-2yl]2,5-diphenyltetrazoliumbromide) (MTT) and DNAse I were obtained from Sigma (St Louis, MO), dimethyl sulphoxide and Proteinase K from Merck (Darmstadt, FRG), BSO from Chemalog (South Plainfield, NJ), nuclease $P_1$ from Boehringer (Mannheim, FRG), ethidium bromide from Serva (Heidelberg, FRG), and $^3$H-thymidine (1 mCi $ml^{-1}$) from New England Nuclear (Boston, MA).

### *Cell lines*

$GLC_4$, a human small cell lung cancer cell line and a CDDP resistant subline, $GLC_4$-CDDP, characterised before by Hospers *et al.* (1988), were cultured in RPMI 1640 medium with 10% heat-inactivated fetal calf serum at 37°C, 5% $CO_2$. $GLC_4$-CDDP was cultured under constant challenge of a monthly dose of 75 μg $ml^{-1}$ CDDP. During the period in which the experiments for this study were performed, doubling times were 16 h and 28 h for $GLC_4$ and $GLC_4$-CDDP, respectively. Total GSH levels were $4.6 \pm 0.5$ and $11.5 \pm 2.4$ μg $mg^{-1}$ protein, respectively in $GLC_4$ and $GLC_4$-CDDP (mean ± s.d.).

### *BSO treatment*

Cells were cultured as described above for 48 h in the presence of 50 μM BSO. At 48 h, GSH was no longer detectable in both cell lines (detection limit: 50 ng), without growth delay or loss of viability. Compared to control cultures no recovery time after BSO pretreatment was needed, after a culture period of 4 days (MTT-assay, described below) no indication of growth delay or loss of viability could be observed.

*GSH*

Cells in the logarithmic phase of growth were harvested 4 days after passage. Cells were washed with ice-cold phosphate buffered saline (PBS) and resuspended in a relevant concentration. All measurements were performed under $V_{max}$ conditions. For cellular GSH determination, cells were resuspended in ice-cold 5% trichloroacetic acid (TCA), mixed vigorously and centrifuged at 4°C (15 min, 10,000 *g*). The supernatant was assayed for total GSH through enzyme recycling under conditions similar to those described by Tietze (1969). The detection limit was 0.1 $\mu g\ mg^{-1}$ protein.

For nuclear GSH, nuclei were isolated according to the method described by Pommier *et al.* (1983). Cells were washed twice with ice-cold PBS and resuspended in a 1/10 volume nucleus buffer (150 mM NaCl, 1 mM $KH_2PO_4$, 5 mM $MgCl_2$, 1 mM EGTA and 0.1 mM dithiothreitol, pH 6.4) at 4°C. Then 9/10 volume of nucleus buffer containing 0.3% Triton X-100 at 4°C was added and the mixture was incubated for 10 min at 4°C. The nuclei were pelleted by centrifugation and resuspended in nucleus buffer at 4°C. Nuclei were examined microscopically after staining with trypan blue to confirm the recovery of nuclei and the absence of cytoplasm. Nuclei were then resuspended in ice-cold 5% TCA, mixed vigorously and centrifuged at 4°C (15 min, 10,000 *g*). The supernatant was assayed for total GSH as described above.

For protein determination the Lowry assay was used (Lowry *et al.*, 1951). All measurements were performed on a Zeiss PMQ spectrophotometer. A minimum of three separate experiments were performed.

*Dynamics of GSH*

The effect of continuous exposure to 5.8 μM CDDP (the dose which inhibits the cell survival of $GLC_4$ by 50% ($IC_{50}$) after 1 h of incubation) on the GSH content of $GLC_4$ and $GLC_4$-CDDP was measured by sampling for GSH and protein determination at different time intervals varying up to 5 h. Also, the effect of continuous exposure to 32 μM CDDP (the $IC_{50}$ of $GLC_4$-CDDP after 1 h incubation) on the GSH content of $GLC_4$-CDDP was measured. A minimum of two separate experiments were performed in duplicate.

*Drug sensitivity assay*

The Microculture tetrazolium assay (MTA) used in this research is based on the cellular reduction of MTT by the mitochondrial dehydrogenase of viable cells to a blue formazan product which can be measured spectrophotometrically (Carmichael *et al.*, 1987). Before the assays were performed, the linear relationship of cell number to MTT formazan crystal formation was checked and cell growth studies were performed. Cells were in the exponential phase of growth at the moment of testing and at least two to three cell divisions should have taken place. Care was taken to select one day (at day 4) to test cell survival for both cell lines. This was possible when we started with 5,000 and 15,000 cells for $GLC_4$ and $GLC_4$-CDDP, respectively.

Incubation of 5,000 cells per well for $GLC_4$ and 15,000 cells per well for $GLC_4$-CDDP proceeded in a total volume of 0.1 ml culture medium with CDDP for 4 h, in 96-well culture plates (Nunc, Gibco, Paisley, UK). For experiments after 48 h BSO incubation, the cells were exposed to CDDP for 4 h in culture medium without BSO. After the CDDP incubation, cells were washed three times by removing the medium after centrifugation of the microtitre plate (10 min, 180 *g*) followed by addition of fresh medium. After a culture period of 4 days, 20 μl of MTT-solution (5 mg MTT $ml^{-1}$ PBS) was added to each well for 3.5 h. Thereafter, the plates were centrifuged (30 min, 180 *g*) and the supernatant was carefully aspirated, so as not to disturb the formazan crystals. Dimethyl sulphoxide 100% (200 μl) was used to dissolve the formazan crystals. The plate was immediately read at 520 nm using a scanning microtitre well spectrophotometer (Titertek Multiskan, Flow Lab, Irvine, UK). The percentage cell survival was calculated by dividing the mean of the test sample by the mean of the untreated sample. Controls consisted of media without cells (background extinction) and cells incubated in wells with medium instead of the drug. At least three separate experiments were performed in quadruplicate at each tested concentration.

*Pt determinations*

The amount of platinum (Pt) was determined with a model 1275 atomic absorption spectrophotometer (AAS) equipped with a model GTA-95 graphite tube atomizer and an autosampler (Varian Techtron Pty Ltd, Mulgrave, Victoria, Australia). A solution of CDDP in the solvent used in each experiment was used for calibration. The Pt detection limit was 2.5 pmol.

*Cellular Pt measurements*

For cellular Pt measurements, $5 \times 10^6$ cells were incubated for 4 h with CDDP concentrations ranging from 83 to 500 μM. After three washes with PBS at 4°C, pellets were dissolved in 0.5 ml 65% $HNO_3$ in an oven at 70°C for 2 h. The amount of Pt was determined as described above. A minimum of three separate experiments were performed at each CDDP incubation concentration.

*Total Pt bound to DNA*

The amount of Pt bound to the DNA was measured after treating $5 \times 10^7$ cells with CDDP concentrations ranging from 83 to 500 μM for 4 h. The cells were washed three times with PBS at 4°C, and the DNA was isolated by using the technique described by Fichtinger-Schepman *et al.* (1987). Briefly, a phenol extraction and ethanol precipitation was followed by RNase treatment. The remaining proteins were extracted by chloroform/iso-amylalcohol. After isolation, the DNA was dissolved 1 N HCl. The DNA content was estimated by absorption at 260 nm; the amount of Pt in the sample was estimated by AAS as described by Roberts and Fraval (1980). At least three independent experiments were performed at each CDDP concentration.

*Pt-GG adduct*

The Pt-GG was measured by AAS after treating the cells with CDDP for 4 h. DNA was isolated from $5 \times 10^7$ cells after CDDP treatment and subsequent washing with PBS, as described earlier. After digestion of DNA, to 350 μl DNA solution in distilled $H_2O$, 39 μl of buffer was added (100 mM Tris-HCl, 40 mM $MgCl_2$, 1 mM $Na_2EDTA$) supplemented with 9.4 μl 10 mM $ZnSo_4$, 14 μl DNAse I (3,000 U $ml^{-1}$) and 39 μl Nuclease $P_1$ (1 mg $ml^{-1}$), the mixture was incubated at 37°C overnight after which 25 μl proteinase K (18 mg $ml^{-1}$) was added. This was followed by incubation for another 2 h at 37°C. Subsequently, the digest was heated for 5 min at 100°C and, after the addition of 20.3 μl 1 M Tris-HCl, centrifuged in an Eppendorf centrifuge for 2 min), the adduct was separated by anion-exchange column chromatography on the Mono Q HR 5/5 column having a particle size of 10 μM (Pharmacia, Uppsala, Sweden) according to the method described by Fichtinger-Schepman *et al.* (1987). The Pt-GG adduct content in the eluate fractions were determined by Pt measurements with AAS. The total DNA content of a sample was estimated by absorption at 260 nm (Fichtinger-Schepman *et al.*, 1985). At least three independent experiments were performed at each CDDP concentration.

*DNA interstrand cross-links (ISC)*

Cells were incubated for 4 h with CDDP concentrations ranging from 83 to 500 μM and washed with PBS at 4°C three times. The pellets were subsequently resuspended and divided into two parts. The amount of ISC formed was measured with the ethidium bromide fluorescence assay, as described by de Jong *et al.* (1986). After lysation for 15 h and the addition of the ethidium bromide, the DNA in one part of each sample was denatured by boiling followed by rapid cooling. The other part was kept at room temperature. The fluorescence of both parts was measured in a Kontron spectrofluorometer (excitation 525 nm, emission 580 nm). The fluorescence in the denatured part is related to the amount of ISC formed. The fluorescence in the other part is related to the total amount of DNA present in that particular sample.

*Repair of DNA-Pt*

The removal of the amount of Pt bound to DNA was measured after incubating $10^8$ cells per sample for 4 h with 33 μM CDDP (the maximal tolerable dose of CDDP without inducing cell toxicity at $t = 22$ h of the repair period). This procedure was followed by harvesting cells for total Pt-DNA binding. After CDDP treatment, half of the sample (repair $t = 0$ h) was washed three times with ice-cold PBS, pelleted by centrifugation and frozen. The other sample was washed twice with medium (37°C) and resuspended in fresh medium for a repair period of 22 h after which the cells were washed, pelleted and frozen as just above. At least three separate experiments, each in duplicate, were performed.

*Correction for dilution by DNA synthesis*

Since the repair of the amount of Pt bound to DNA was quantified per mg of isolated DNA, correction for dilution by DNA synthesised during the 22 h post-treatment incubated period was carried out according to the method described by Bedford *et al.* (1988). Cells were labelled for 44 h with 20 nCi $ml^{-1}$ $^3$H-thymidine followed by 4 h in isotope-free medium. Cells were then exposed for 4 h to 33 μM CDDP, as described previously, and harvested either immediately or after 22 h. DNA was extracted by heating the cell pellets at 70°C for 1 h in 1 N perchloric acid. Radioactivity was determined in 200 μl aliquots of supernatant and the DNA content of the remainder was estimated spectrophotometrically as described above. The dilution factor was calculated as the specific activity of DNA at 22 h divided by the specific activity of DNA at 0 h. The apparent amount of Pt bound to DNA at 22 h divided by the dilution factor gave the amount of Pt bound to DNA.

*Statistics*

Statistical significance was determined with the unpaired Student's $t$ test and for the BSO experiments with the paired Student's $t$ test. $P$ values $<0.05$ were considered significant.

### Results

Survival curves of $GLC_4$ and $GLC_4$-CDDP after 50 μM BSO for 48 h showed an increased CDDP induced cytotoxicity after 4 h of CDDP treatment (Figure 1). $IC_{50}$ values were 1.0 μM and 22 μM for $GLC_4$ and $GLC_4$-CDDP respectively and decreased to 0.6 μM and 7.8 μM respectively. BSO modulated CDDP survival in the sensitive cell line by a factor of 1.7 and in the CDDP resistant cell line by a factor of 2.8. Dose modifying factors (DMF) were calculated at the $IC_{10}$, $IC_{20}$, ..., $IC_{90}$ for both cell lines to confirm a significantly higher BSO modulating effect on the CDDP induced cytotoxicity in $GLC_4$-CDDP compared to $GLC_4$. DMF, obtained from the separate survival curves, were $1.90 \pm 0.63$ ($n = 21$) and $3.03 \pm 1.51$ ($n = 26$) in $GLC_4$ and $GLC_4$-CDDP respectively ($P < 0.0025$).

**Figure 1** Effect of pretreatment of $GLC_4$ (O——O) and $GLC_4$-CDDP (●——●) with 50 μM BSO for 48 h on CDDP induced cytotoxicity, after 4 h incubation with CDDP, as measured by MTA, without BSO (——) and following pretreatment with BSO (– – –) ($n \geq 4$, bars = s.e.m.). For $GLC_4$ significantly different values were observed at 0.25 μM, $P < 0.0005$; at 0.5 μM, $P < 0.0005$; at 1.0 μM, $P < 0.0005$; at 5.0 μM, $P < 0.025$, and at 10 μM, $P < 0.0005$. For $GLC_4$-CDDP at 1.0 μM, $P < 0.05$; at 5.0 μM, $P < 0.0005$; at 10 μM, $P < 0.0005$; at 50 μM, $P < 0.0005$; at 100 μM, $P < 0.0005$; and at 250 μM, $P < 0.025$.

Nuclear GSH contents were $89 \pm 12$ ng $mg^{-1}$ cellular protein and $235 \pm 138$ ng $mg^{-1}$ cellular protein (mean ± s.d., $n = 3$) in $GLC_4$ and $GLC_4$-CDDP respectively ($P < 0.05$). The elevated nuclear GSH in $GLC_4$-CDDP compared to $GLC_4$ is proportional to the elevated cellular GSH in $GLC_4$-CDDP compared to $GLC_4$.

Figure 2 shows the effect of continuous exposure to 5.8 μM

**Figure 2** Effect of continuous exposure to 5.8 μM CDDP on the GSH content of $GLC_4$ (O) and $GLC_4$-CDDP (■) and the effect of continuous exposure to 32 μM CDDP on the GSH content of $GLC_4$-CDDP (●) ($n = 2$, in duplicate).

CDDP on the GSH content of $GLC_4$ and $GLC_4$-CDDP, and the effect of continuous exposure to 32 µM CDDP on the GSH content of $GLC_4$-CDDP. Continuous exposure to CDDP led to an increase in GSH content in $GLC_4$. The GSH in these cells was doubled after 5 h and contained 80% of the amount normally (without CDDP exposure) present in $GLC_4$-CDDP. A short period of increase of the GSH level was noted in $GLC_4$-CDDP followed by a steady state at 70–90% of the initial GSH level at both 5.8 and 32 µM CDDP.

No difference in the cellular Pt content between $GLC_4$ and $GLC_4$-CDDP was observed after correction for cellular protein or cellular volume (Hospers *et al.*, 1988). The pretreatment of cells with BSO did not alter the cellular Pt content in $GLC_4$ and $GLC_4$-CDDP after 4 h CDDP treatment as measured by AAS (data not shown).

The amount of Pt-DNA binding was significantly lower in $GLC_4$-CDDP as compared to $GLC_4$. The effect of pretreatment with BSO on the Pt-DNA binding after 4 h CDDP treatment is shown in Figure 3. Following BSO pretreatment, an increased Pt-DNA binding for $GLC_4$-CDDP was seen whereas the Pt-DNA binding in $GLC_4$ remained the same. In $GLC_4$-CDDP a reduced number of ISC was observed as compared to $GLC_4$. Figure 4 shows the effect of pretreatment with BSO on the ISC formation. Although an increase in ISC seemed to appear in $GLC_4$-CDDP after BSO pretreatment, no significant change in formation could be confirmed. The initial decreased formation of ISC in the resistant cell line when compared to the sensitive line could be eliminated. The effect of pretreatment with BSO on the formation of the Pt-GG adduct is shown in Figure 5. A significant increase in Pt-GG adduct formation (2.6-fold) after pretreatment with BSO was observed in $GLC_4$-CDDP whereas the Pt-GG adduct formation after pretreatment with BSO remained the same for $GLC_4$.

Table I shows the repair capacity of $GLC_4$ and $GLC_4$-CDDP after incubation for 4 h with 33 µM CDDP followed by a drug-free culture period of 22 h. Both $GLC_4$ and $GLC_4$-CDDP showed a significant reduction in the amount of Pt bound to DNA after the 22 h drug-free culture period. Pretreatment with 50 µM BSO for 48 h annihilated this repair in both cell lines.

**Figure 4** Effect of pretreatment of $GLC_4$ (O——O) and $GLC_4$-CDDP (●——●) with 50 µM BSO for 48 h on the ISC formation after 4 h incubation with CDDP, without BSO (——) and following BSO pretreatment (– – –) ($n \geq 6$, bars = s.e.m.). Significantly different values were observed for $GLC_4$-CDDP versus $GLC_4$ at all tested concentrations: at 83 µM, $P < 0.005$; at 167 µM, $P < 0.005$; at 333 µM, $P < 0.025$; and at 500 µM, $P < 0.0005$. No significant changes were observed in either cell line following BSO treatment.

**Figure 3** Effect of pretreatment of $GLC_4$ (O——O) and $GLC_4$-CDDP (●——●) with 50 µM BSO for 48 h on the Pt-DNA binding after 4 h incubation with CDDP, without BSO (——) and following BSO treatment (– – –) ($n \geq 3$, bars = s.e.m.). Significantly different values were observed for $GLC_4$-CDDP versus $GLC_4$ at all tested concentrations: at 83 µM, $P < 0.05$; at 167 µM, $P < 0.01$; at 333 µM, $P < 0.0025$; and at 500 µM, $P < 0.0025$. Significantly different values were observed for $GLC_4$-CDDP versus $GLC_4$-CDDP + BSO: at 333 µM, $P < 0.05$; and at 500 µM, $P < 0.05$. Significantly different values for $GLC_4$-CDDP + BSO versus $GLC_4$ were only observed at 83 µM, $P < 0.01$; and at 333 µM, $P < 0.05$.

**Figure 5** Effect of pretreatment of $GLC_4$ (O——O) and $GLC_4$-CDDP (●——●) with 50 µM BSO for 48 h on the formation of the Pt-GG adduct after 4 h incubation with CDDP, without BSO (——) and following BSO pretreatment (– – –) ($n \geq 3$, bars = s.e.m.). Significantly different values for $GLC_4$-CDDP versus $GLC_4$-CDDP + BSO were observed at 167 µM, $P < 0.025$; at 333 µM, $P < 0.0125$; and at 500 µM, $P < 0.05$. A significantly different value for $GLC_4$-CDDP + BSO versus $GLC_4$ was observed at 333 µM, $P < 0.05$.

**Table I** Repair of Pt bound to DNA, after 4 h exposure to 33 μM CDDP

| | t (h) | fmol Pt μg$^{-1}$ DNA |
|---|---|---|
| $GLC_4$ | 0 | 475 ± 84[a] |
| | 22 | 380 ± 47[b] |
| $GLC_4$-CDDP | 0 | 289 ± 65[e] |
| | 22 | 168 ± 82[c,f] |
| $GLC_4$ + BSO | 0 | 451 ± 46[h] |
| | 22 | 420 ± 43[d,h] |
| $GLC_4$-CDDP + BSO | 0 | 283 ± 107[g,h] |
| | 22 | 261 ± 102[d,g,h] |

[a]Mean ± s.d., $n = 3$, in duplicate. [b]$P < 0.025$, $t = 0$ h versus $t = 22$ h. [c]$P < 0.01$, $t = 0$ h versus $t = 22$ h. [d]Not significant, $t = 0$ h versus $t = 22$ h. [e]$P < 0.0025$ $GLC_4$-CDDP $t = 0$ h versus $GLC_4$ $t = 0$ h [f]$P < 0.0005$ $GLC_4$-CDDP $t = 22$ h versus $GLC_4$ $t = 22$ h. [g]$P < 0.005$ $GLC_4$-CDDP + BSO $t = 0$ h versus $GLC_4$ + BSO $t = 0$ h. $GLC_4$-CDDP + BSO $t = 22$ h versus $GLC_4$ + BSO $t = 22$ h. [h]NS $GLC_4$ $t = 0$ h versus $GLC_4$ + BSO $t = 0$ h. $GLC_4$ $t = 22$ h versus $GLC_4$ + BSO $t = 22$ h. $GLC_4$-CDDP $t = 0$ h versus $GLC_4$-CDDP + BSO $t = 0$ h. $GLC_4$-CDDP $t = 22$ h versus $GLC_4$-CDDP + BSO $t = 22$ h.

### Discussion

Given the sulphydryl reactive properties of CDDP, alterations in cellular thiol content may be a defence mechanism generated in CDDP resistant cells. GSH, the main non-protein thiol present in cells, has been shown to be an important determinant of the sensitivity of cells to a wide variety of drugs including cytostatic agents (Arrick & Nathan, 1984). Elevated levels of GSH have been associated with drug-resistant phenotypes developed in cells exposed to a number of electrophilic drugs (Hamilton *et al.*, 1985; Green *et al.*, 1984; Tan *et al.*, 1987; Evans *et al.*, 1987). Moreover, cells with elevated levels of GSH also appeared to be resistant to CDDP (Richon *et al.*, 1987; Waud, 1987; Hamilton *et al.*, 1985; Hromas *et al.*, 1987; Behrens *et al.*, 1987). However, when BSO is used to deplete GSH levels, the effect on CDDP induced cytotoxicity varied in the different studies. In the cell lines used in this study, the CDDP resistant subline $GLC_4$-CDDP had a 2.5-fold increase in GSH content as compared to the CDDP sensitive cell line. Exposure of the cells to BSO reduced the cellular GSH content to a non-detectable level in both cell lines and increased the CDDP induced cytotoxicity. This is in agreement with the findings of Hamilton *et al.* (1985) and Hromas *et al.* (1987). In other studies, however, no effect of BSO on CDDP-induced cytotoxicity was found (Teicher *et al.*, 1987; Richon *et al.*, 1987).

The exact mechanism by which GSH influences CDDP cytotoxicity is not yet completely clear. CDDP can be inactivated by direct binding to the sulphydryl moiety of GSH, thereby preventing the drug from reaching the critical DNA. This cytosolic inactivation should take place during the limited time prior to reaction with DNA.

The steady state of GSH is generally used to express the capacity of this defence system, although the kinetics of the GSH status should give the more dynamic representation of the continuous availability of this defence. In the present study the GSH status was depicted under continuous pressure of CDDP. $GLC_4$-CDDP was able to maintain its elevated GSH level under pressure of both 5.8 and 32 μM CDDP suggesting an enhanced GSH biosynthetic capacity in $GLC_4$-CDDP. $GLC_4$ showed a fast synthesis of GSH under pressure of 5.8 μM CDDP. After continuous incubation for 5 h with CDDP, the GSH level reached about twice its own original level, which corresponds to 80% of the GSH level of $GLC_4$-CDDP (Figure 2). In the resistant cells, however, more GSH was available throughout the observation period. It is evident that exposure to CDDP can increase the amount of GSH (or can induce GSH synthesis) in some cells. Thus, treatment with BSO and subsequent exposure to CDDP might not lead to unmeasurably low levels of GSH in such cells.

No change in cellular Pt content in both cell lines was measured following CDDP exposure to BSO pretreated and control cells, thus eliminating an effect of BSO on membrane permeability.

The role of GSH at the nuclear level is still unclear. Eastman (1987) showed that GSH can react *in vitro* with monofunctional adducts of platinated DNA and prevent them from rearranging to form toxic bifunctional adducts. The formation and protective role of this GSH-adduct is, however, still unclear.

Only small amounts of GSH could be detected in the nuclei of $GLC_4$ and $GLC_4$-CDDP. The elevation of nuclear GSH in $GLC_4$-CDDP as compared to $GLC_4$ is in proportion to the increased cellular GSH content of this cell line. Since some leakage of GSH can be assumed to occur from the nuclei during the isolation procedure, the nuclear GSH values are probably underestimated. However, Edgren and Révész (1987) also detected less than 1% of the total GSH in the nuclei of Chinese hamster V79-379A cells. GSH depletion by BSO was also demonstrated by them to be more efficient in whole cells than in isolated nuclei for a period varying in length up to 18 h. In the present study no GSH could be detected in the nuclei of either $GLC_4$ and $GLC_4$-CDDP after a 48 h pretreatment with BSO.

ISC have been reported to be enhanced after BSO-induced GSH depletion in human leukaemia cells insensitive to activated cyclophosphamide (Crook *et al.*, 1986) as well as human melanoma cells, which generally have a high degree of inherent resistance to bifunctional alkylating agents (Hansson *et al.*, 1988). An effect of BSO on the induction of sister chromatid exchanges has also been published (Evans *et al.*, 1987). In this study, neither changes in the amount of Pt bound to DNA after GSH depletion, nor changes in the formation of ISC or Pt-GG adducts were observed in $GLC_4$. In GSH depleted $GLC_4$-CDDP cells, however, an increase in the amount of Pt bound to DNA was found. The main Pt-containing intrastrand cross-link in digested DNA, the Pt-GG adduct, also increased after GSH depletion. The difference in formation of ISC between $GLC_4$ and $GLC_4$-CDDP was annihilated by pretreatment with BSO (Figures 3–5).

Lai *et al.* (1989) recently described that, aside from the generally considered BSO effects on alkylating agents related to GSH reduction, BSO treatment partially inhibited DNA repair after CDDP damage in an ovarian cancer cell line with *in vitro* induced resistance to CDDP, as measured by unscheduled DNA synthesis.

In the present study, the repair of the amount of Pt bound to DNA was assayed at an incubation concentration of 33 μM CDDP for 4 h. Under these conditions, repair of the amount of Pt bound to DNA could be observed in both $GLC_4$ and $GLC_4$-CDDP, a capacity which was earlier described to be only operational in the $GLC_4$-CDDP line with a resistance factor of 11 (Hospers *et al.*, 1990), most probably due to the treatment schedule ($t = 2$ h compared to $t = 4$ h) in combination with a high CDDP concentration (especially for $GLC_4$) used in the previous study. After GSH depletion, both cell lines were unable to repair the CDDP induced amount of Pt bound to DNA. The elimination of the quenching of monofunctional DNA adducts by GSH, resulting from BSO pretreatment, may be an explanation for the loss of repair capacity in both cell lines.

A disparity in concentrations used for the various parameters due to the detection limit for Pt as measured by AAS was unavoidable. Taking this into account, we conclude that an increased GSH level and GSH synthesis capacity was demonstrated in CDDP resistant cells. The observations after BSO treatment, namely the 2.6-fold increase in the CDDP induced cytotoxicity in $GLC_4$-CDDP, suggest two roles for GSH in CDDP resistance, namely a cytosolic elimination resulting in less DNA platination, and a more pronounced nuclear effect on the formation and repair of DNA platinum adducts. The still remaining thirteen-fold resistance after BSO treatment in these CDDP resistant cell lines, however, should dispute the 'relative' importance of this GSH

mediated CDDP resistance in perspective with the other mechanisms that are operative in this line, such as a decreased total DNA platination, a decreased Pt-GG adduct formation, a decreased ISC formation and/or a more efficient DNA repair.

The authors wish to thank H. Bloemhof and G.J. Meersma for technical assistance. This study was supported in part by a grant from the Dutch Cancer Foundation (GUKC 86-01).

### References

ANDREWS, P.A., VELURY, S., MANN, S.C. & HOWELL, S.B. (1988). *Cis*-diammine-dichloroplatinum (II) accumulation in sensitive and resistant human ovarian carcinoma cells. *Cancer Res.*, **48**, 68.

ARRICK, B.A. & NATHAN, C.F. (1984). Glutathione metabolism as a determinant of therapeutic efficacy: a review. *Cancer Res.*, **44**, 4224.

BEDFORD, P., FICHTINGER-SCHEPMAN, A., SHELLARD, S.A., CLAIRE WALKER, M., MASTERS, J.R.W. & HILL, B.T. (1988). Differential repair of platinum-DNA adducts in human bladder and testicular tumor continuous cell lines. *Cancer Res.*, **48**, 3019.

BEHRENS, B.C., HAMILTON, T.C., MASUDA, H. & 7 others (1987). Characterization of a *cis*-diamminedichloroplatinum(II)-resistant human ovarian cancer cell line and its use in evaluation of platinum analogues. *Cancer Res.*, **47**, 414.

CARMICHAEL, J., DE GRAFT, W.G., GAZDAR, A.F., MINNA, I.D. & MITCHELL, J.B. (1987). Evaluation of tetrazolium-based semiautomated colorimetric assay: assessment of chemosensitivity testing. *Cancer Res.*, **47**, 936.

CROOK, T.R., SOUHAMI, R.L., WHYMAN, G.D. & MCLEAN, A.E.M. (1986). Glutathione depletion as a determinant of sensitivity of human leukemia cells to cyclophosphamide. *Cancer Res.*, **46**, 5035.

DE JONG,, S., ZIJLSTRA, J.G., TIMMER-BOSSCHA, H., MULDER, N.H. & DE VRIES, E.G.E. (1986). Detection of DNA cross-links in tumor cells with the ethidium bromide fluorescence assay. *Int. J. Cancer*, **37**, 557.

EASTMAN, A. (1987). Cross-linking of glutathione to DNA by cancer chemotherapeutic platinum coordination complexes. *Chem. Biol. Interactions*, **61**, 241.

EASTMAN, A. & SCHULTE, N. (1988). Enhanced DNA repair as a mechanism of resistance to *cis*-diamminedichloroplatinum(II). *Biochemistry*, **27**, 4730.

EDGREN, M. & RÉVÉSZ, L. (1987). Compartmentalised depletion of glutathione in cells treated with buthionine sulphoximine. *Br. J. Radiol.*, **60**, 723.

EVANS, C.G., BODELL, W.J., TOKUDA, K., DOANE-SETZER, P. & SMITH, M.M. (1987). Glutathione and related enzymes in rat brain tumor cell resistance to 1,3-*bis*(2-chloroethyl)-1-nitrosourea and nitrogen mustard. *Cancer Res.*, **47**, 2525.

FICHTINGER-SCHEPMAN, A.M.J., BAAN, R.A., LUITEN-SCHUITE, A., VAN DIJK, M. & LOHMAN, P.H.M. (1985). Immunochemical quantitation of adducts induced in DNA by *cis*-diammine-dichloroplatinum (II) and analysis of adduct-related DNA-unwinding. *Chem. Biol. Interactions*, **55**, 275.

FICHTINGER-SCHEPMAN, A.M.J., VAN OOSTEROM, A.T., LOHMAN, P.H.M. & BERENDS, F. (1987). Cisplatin-induced DNA adduct in peripheral leukocytes from seven patients: quantitative immunochemical detection of the adduct induction and removal after a single dose of cisplatin. *Cancer Res.*, **47**, 3000.

GREEN, J.A., VISTICA, D.T., YOUNG, R.C., HAMILTON, T.C., ROGAN, A.M. & OZOLS, R.F. (1984). Potentiation of melphalan cytotoxicity in human ovarian cancer cell lines by glutathione depletion. *Cancer Res.*, **44**, 5427.

HAMILTON, T.C., WINKER, M.A., LOUIE, K.G. & 7 others (1985). Augmentation of adriamycin, melphalan and cisplatin cytotoxicity in drug-resistant and -sensitive human ovarian carcinoma cell lines by buthionine sulfoximine mediated glutathione depletion. *Biochem. Pharmacol.*, **34**, 2583.

HANSSON, J., EDGREN, M., EHRSSON, H., RINGBORG, U. & NILSSON, B. (1988). Effect of D,L-buthionine-S,R-sulfoximine on cytotoxicity and DNA cross-linking induced by bifunctional DNA-reactive cytostatic drugs in human melanoma cells. *Cancer Res.*, **48**, 19.

HOSPERS, G.A.P., MULDER, N.H., DE JONG, B. & 5 others (1988). Characterization of a human small cell lung carcinoma cell line with acquired cisplatin resistance *in vitro*. *Cancer Res.*, **48**, 6803.

HOSPERS, G.A.P., DE VRIES, E.G.E. & MULDER, N.H. (1990). The formation and removal of cisplatin (CDDP) induced DNA adducts in a CDDP sensitive and resistant human small cell lung carcinoma (hSCLC) cell line. *Br. J. Cancer.*, **61**, 79.

HROMAS, R.A., ANDREWS, P.A., MURPHY, M.P. & BURNS, C.P. (1987). Glutathione depletion reverses cisplatin resistance in murine L1210 leukemia cells. *Cancer Lett.*, **34**, 9.

KRAKER, A.J. & MOORE, C.W. (1988). Accumulation of *cis*-diamminedichloroplatinum(II) and platinum analogues by platinum-resistant murine leukemia cells *in vitro*. *Cancer Res.*, **48**, 9.

LAI, G.M., OZOLS, R.F., YOUNG, R.C. & HAMILTON, T.C. (1989). Effect of glutathione on DNA repair in cisplatin-resistant human ovarian cancer cell lines. *J. Natl Cancer Inst.*, **81**, 535.

LOWRY, O.H., ROSEBROUGH, N.J., FARR, A.L. & RANDALL, R.J. (1951). Protein measurement with the folin-phenol reagent. *J. Biol. Chem.*, **193**, 265.

MICETICH, K., ZWELLING, L.A. & KOHN, K.W. (1983). Quenching of DNA: platinum (II) monoadducts as a possible mechanism of resistance to *cis*-diamminedichloroplatinum (II) in L1210 cells. *Cancer Res.*, **43**, 3609.

POMMIER, Y., ZWELLING, L.A., MATTERN, M.R. & 4 others (1983). Effect of dimethyl sulfoxide and thiourea upon intercalator-induced DNA single-strand breaks in mouse leukemia (L1210) cells. *Cancer Res.*, **43**, 5718.

RICHON, V.M., SCHULTE, N. & EASTMAN, A. (1987). Multiple mechanisms of resistance to *cis*-diamminedichloroplatinum (II) in murine leukemia L1210 cells. *Cancer Res.*, **47**, 2056.

ROBERTS, J.J. & FRAVAL, H.N.A. (1980). Repair of *cis*-platinum (II) diammine dichloride-induced DNA damage and cell sensitivity. In *Cisplatin, Current Status and New Developments*, Prestayko, A.W., Crooke, S.T. & Carter, S.K. (eds) p. 57. Acadmic Press: New York.

TAN, K.B., MATTERN, M.R., BOYCE, R.A. & SCHEIN, P.S. (1987). Elevated DNA topoisomerase II activity in nitrogen mustard-resistant human cells. *Proc. Natl Acad. Sci. USA*, **84**, 7668.

TEICHER, B.A., HOLDEN, S.A., KELLEY, M.J. & 5 others (1987). Characterization of a human squamous carcinoma cell line resistant to *cis*-diamminedichloro-platinum (II). *Cancer Res.*, **47**, 388.

TIETZE, F. (1969). Enzymic method for quantitative determination of nanogram amounts of total and oxidized glutathione. *Anal. Biochem.*, **27**, 502.

WAUD, W.R. (1987). Differential uptake of *cis*-diammine-dichloroplatinum(II) by sensitive and resistant murine L1210 leukemia cells. *Cancer Res.*, **47**, 6549.